# Chiroptical Metasurfaces: Principles, Classification, and Applications

**DOI:** 10.3390/s21134381

**Published:** 2021-06-26

**Authors:** Joohoon Kim, Ahsan Sarwar Rana, Yeseul Kim, Inki Kim, Trevon Badloe, Muhammad Zubair, Muhammad Qasim Mehmood, Junsuk Rho

**Affiliations:** 1 Department of Mechanical Engineering, Pohang University of Science and Technology, Pohang 37673, Korea; kimjuhoon@postech.ac.kr (J.K.); yesl3651@postech.ac.kr (Y.K.); inki93@postech.ac.kr (I.K.); trevon@postech.ac.kr (T.B.); 2 NanoTech Lab, Department of Electrical Engineering, Information Technology University of the Punjab, Ferozepur Road, Lahore 54600, Pakistan; ahsan.sarwar@itu.edu.pk (A.S.R.); muhammad.zubair@itu.edu.pk (M.Z.); 3 Department of Chemical Engineering, Pohang University of Science and Technology, Pohang 37673, Korea

**Keywords:** chiral sensing, metamaterial, metasurface, chiroptical metamaterial, chiroptical metasurface, sensing, circular dichroism (CD), optical rotatory dispersion (ORD), metahologram, metalens, multiplexing metahologram, multifunctional metahologram, multiplexing metalens, multifunctional metalens

## Abstract

Chiral materials, which show different optical behaviors when illuminated by left or right circularly polarized light due to broken mirror symmetry, have greatly impacted the field of optical sensing over the past decade. To improve the sensitivity of chiral sensing platforms, enhancing the chiroptical response is necessary. Metasurfaces, which are two-dimensional metamaterials consisting of periodic subwavelength artificial structures, have recently attracted significant attention because of their ability to enhance the chiroptical response by manipulating amplitude, phase, and polarization of electromagnetic fields. Here, we reviewed the fundamentals of chiroptical metasurfaces as well as categorized types of chiroptical metasurfaces by their intrinsic or extrinsic chirality. Finally, we introduced applications of chiral metasurfaces such as multiplexing metaholograms, metalenses, and sensors.

## 1. Introduction

Understanding and analyzing a chiral response of molecules or particles is crucial for research in imaging and spectroscopy. Research for detecting the chiral properties of chiral objects has been reported, but the weak chiroptical signal is a critical limitation in sensing with natural chiral materials. Thanks to the progress in nanoscience and nanofabrication technologies, we can design and achieve nanoscale or micrometerscale chiral building blocks that amplify the chiral response. Metamaterials [1,2], consisting of periodic subwavelength artificial structures known as meta-atoms, have attracted significant attention due to their remarkable ability to modulate electromagnetic (EM) waves. Metamaterials enable exceptional properties that cannot be achieved with natural materials, such as negative refractive index [3,4], zero-index materials [5], and ultra-high-index materials [6,7]. 

Metasurfaces [8,9,10], the two-dimensional (2D) counterpart of metamaterials with subwavelength thicknesses, have been researched as EM wavefront shaping thin-flat optical devices. Moreover, metasurfaces are much easier to fabricate due to their planar nature. To fully take advantage of metasurfaces, various applications have been studied, such as metaholograms [11,12,13,14,15,16,17,18,19,20], metalenses [21,22,23,24], absorbers [25,26,27], structural colors [28,29,30,31,32,33,34,35,36], beam splitters [37,38,39], optical diodes [40,41], LiDAR applications [42,43], wide bandwith antennas [44,45,46,47,48,49,50], on-chip antennas [51,52,53], and MIMO and SAR antennas [54,55,56]. In particular, due to their ability to enhance chiral signals by field localization, chiral material sensing based on metasurfaces has sparked significant attention in recent years. 

Chiral metamateirlas and metasurfaces [57,58,59,60,61,62,63,64,65,66], consisting of meta-atoms that lack mirror symmetry, have been actively researched due to their differing exotic optical properties when interacting with left (LCP) or right circularly polarized (RCP) light. They can exhibit chiroptical phenomena, such as the difference in the propagation velocity, known as optical rotatory dispersion (ORD), and the difference of absorption, known as circular dichroism (CD), between LCP and RCP light. Therefore, the chiroptical metasurfaces have attracted interest for the analysis of enantiomers such as biomolecules, protein structures, the electronic transitions of molecules, and the conformation of small molecules [67,68,69].

In this paper, we reviewed the fundamentals of chiroptical metasurfaces and introduced various applications of them. In Section 2, we introduced fundamentals of chiroptical metasurfaces. We explained how the local fields produced by metasurfaces interact with chiral molecules to enhance the chiroptical signal. In Section 3, we categorized types of chiroptical metasurfaces into intrinsic and extrinsic chirality. The meta-atoms of intrinsic chiral metasurfaces have chirality-dependent geometries, whereas the meta-atoms of extrinsic chiral metasurfaces are achiral and homogeneous nanostructures. In Section 4, we introduced practical applications of chiroptical metasurfaces. In addition to sensing, chiroptical metasurfaces can also be applied to multiplexing metasurfaces, increasing the amount of information that can be displayed from a single metasurface.

## 2. Fundamentals of Chiroptical Metasurfaces

Chirality describes objects that cannot be superposed by their mirror image through a single rotational or translational operation. Various natural systems such as molecules, DNA, and proteins possess chiral characteristics, and isomers of opposite handedness, called enantiomers, induce distinct optical chirality when they interact with circularly polarized light [70,71]. Examples of these chirality-related phenomena are CD [60,72,73,74], circular birefringence (CB) [75,76,77], the repulsive Casimir force [78], and the spin Hall effect [79]. 

A structure needs to break reflection and rotational symmetries to be considered chiral. To evaluate these conditions, Jones matrices are used with the assumption that the incident plane waves contain a single wavelength/frequency of light (monochromatic) and are also identical in terms of their waveform and phase difference (i.e., they are coherent). A transmission matrix (*T^f^*) that relates the complex incident (*I_x_*_/*y*_) and transmission (*T_x_*_/*y*_) amplitudes of light can then be written as [80].

(1) $$\left[\begin{array}{c} T_{x}\\ T_{y} \end{array}\right]=\left[\begin{array}{cc} t_{xx} & t_{xy}\\ t_{yx} & t_{yy} \end{array}\right]\left[\begin{array}{c} I_{x}\\ I_{y} \end{array}\right]=\left[\begin{array}{cc} A & B\\ C & D \end{array}\right]\left[\begin{array}{c} I_{x}\\ I_{y} \end{array}\right]=T^{f}\left[\begin{array}{c} I_{x}\\ I_{y} \end{array}\right].$$

The terms *t_xx_*, *t_xy_*, *t_yx_*, and *t_yy_* represent the transmission coefficients. The subscript indicates the polarization of the incident (right) and transmitted (left) wave (i.e., *t_xy_* denotes the transmission coefficient of y-polarized incidence and x-polarized transmission). The superscript of transmission matrix (*T^f^*) represents the direction of wave propagation to be forward propagating. The matrix of transmission coefficients can then be employed alongside a rotation matrix *D_r_* to investigate the symmetry conditions by finding a new transmission matrix (*T_r_*).

(2) $$T_{r}=D_{r}^{-1}T^{f}D_{r}=\left[\begin{array}{cc} cos\theta & -sin\theta \\ sin\theta & cos\theta \end{array}\right]\left[\begin{array}{cc} A & B\\ C & D \end{array}\right]\left[\begin{array}{cc} cos\theta & sin\theta \\ -sin\theta & cos\theta \end{array}\right].$$

For a chiral structure, all elements in the transmission matrix above are different. Equations (1) and (2) are in the Cartesian basis, and a transmission matrix ($T_{cir}^{f}$) can be calculated by converting it to a circular basis as,

(3) $$T_{cir}^{f}=\left[\begin{array}{cc} A+D+i\left(B-C\right) & A-D-i\left(B+C\right)\\ A-D+i\left(B+C\right) & A+D-i\left(B-C\right) \end{array}\right]=\left[\begin{array}{cc} t_{++} & t_{+-}\\ t_{-+} & t_{—} \end{array}\right],$$

where “—” represents LCP waves, and “+” represents RCP waves, so too does *t*_+−_ denote LCP incidence and RCP transmission. The difference in transmission between two cross-polarized waves, or asymmetric transmission (AT), can be calculated by the matrix elements (1,2) and (2,1) in Equation (1) (for linear polarization) and Equation (3) (for circular polarization) as shown below.

(4) $$AT_{linear}^{x}={\left(t_{yx}\right)}^{2}-{\left(t_{xy}\right)}^{2}=T_{yx}-T_{xy}=AT_{linear}^{y}$$

(5) $$AT_{circular}^{-}={\left(t_{+-}\right)}^{2}-{\left(t_{-+}\right)}^{2}=T_{+-}-T_{-+}=AT_{circular}^{+}$$

**Figure 1:**
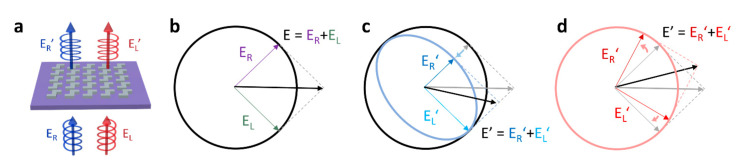
Schematics of chiroptical phenomena: (**a**) Example scheme of chiroptical metasurface. After the incident light interacts with the nanostructures on the metasurface, the amplitude and direction of light can be changed. (**b**) Linearly polarized light (E) can be decomposed into RCP (E_R_) and LCP (E_L_) light with equal amplitudes. (**c**) CD: the amplitudes of the decomposed components vary depending on the absorption. (**d**) CB: the direction of the decomposed components rotates. The resulting light is represented as E’. (**b**–**d**) are modified from reference [69].

The subwavelength scale of nanostructures on the metasurface, called meta-atoms, will typically perform as waveguides or optical antennas. Incident light passes through each meta-atom and is scattered through an optical response, so the resulting light has a different phase and amplitude compared to the initial light. By engineering meta-atoms, optical wavefront can be modulated. The meta-atoms of the chiroptical metasurface induce a drastic difference of optical response depending on whether the incident light is RCP or LCP. As an example, as shown in Figure 1a, if the meta-atoms possess chiral geometry, the optical properties of transmitted light will be different from that of incident light.

CD is defined as the difference of absorption (or transmission) coefficients for the two opposite circularly polarized states of light. All linearly polarized states of light can be decomposed into RCP and LCP states with the same magnitude (Figure 1b). Therefore, if linear polarized light interacts with a chiral object that demonstrates CD, the polarization of the reflected or transmitted light is transformed into an ellipsoidal state (Figure 1c). 

CB, which is also known as optical rotation (OR), signifies that the direction of the decomposed polarized lights is rotated without any change of amplitude. Because the amplitude of each component is invariant, but the rotation angle is different, the resulting light can differ in magnitude from the incident light (Figure 1d). The amount of rotation also varies on the wavelength of the incident light due to the dispersive characteristics of the refractive index that affects the propagation velocity in the medium. The variation of rotation of polarized light depending on wavelength is defined as ORD [81,82].

## 3. Types of Chiroptical Metasurfaces

Chiral responses from natural materials are generally too small to detect easily, so metasurfaces have emerged as an effective way of enhancing chiroptical signals and as chirality-dependent optical devices. By removing one dimension of freedom from 3D metamaterials, 2D chiroptical metasurfaces gain advantages in terms of ease of fabrication, but limit the degree of freedom to break symmetry. Nevertheless, chiroptical metasurfaces have been applied to biological monitoring [71,83], analytic chemistry [84,85,86], and plasmonic sensing [87,88] because they have potential benefits, not only to increase the chiroptical signal by focusing the local fields at specific regions, but also to demonstrate flat, ultrathin, and compact sensing platforms. In this section, we introduced the concepts of intrinsic or extrinsic chirality [88]. 

### 3.1. Intrinsic Chiral Metasurfaces

**Figure 2:**
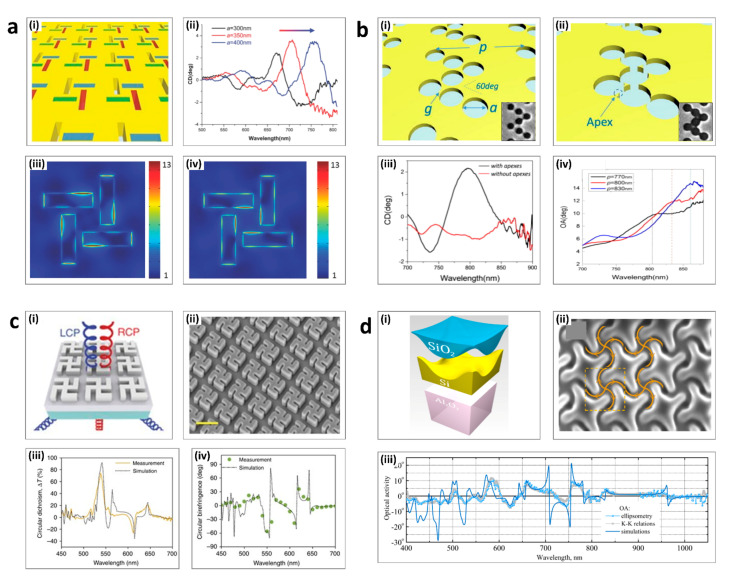
Intrinsic chiral metasurfaces: (**a**) Rectangular slits; (i) Schematic of the metasurface. (ii) The CD spectrum. (iii–iv) The electric field intensity map under RCP and LCP illumination. (**b**) Chiral arrays of achiral nanoholes; (i) The holes are separated by nanoscale gaps (ii) The holes are overlapped to make an apex. (iii) The CD spectrum. (iv) The OA spectrum. (**c**) Gammadion patterns; (i) Schematic of the input and output light and nanostructure. (ii) Scanning electron micrographic (SEM) image. Scale bar = 500 nm. (iii) The CD spectrum (iv) The CB spectrum. (**d**) Four-fold-rotationally-symmetric grooves. (i) Schematic of layers. (ii) SEM image. (iii) The theoretical and simulated OA. (**a**) is adapted from reference [90]. (**b**) is adapted from reference [91]. (**c**) is adapted from reference [92]. (**d**) is adapted from reference [93].

Intrinsic chiral metasurfaces have arrays of meta-atoms that have chirality-dependent geometry or structures [77,88,89]. When the size of the chiral meta-atoms matches the wavelength of the incident light, the chiroptical signal gets stronger as the chirality-dependent interactions are induced even at normal incidence. To enhance chiroptical responses in the visible regime, the meta-atoms should interact with visible wavelengths of light, meaning that sophisticated nanofabrication techniques are required. Intrinsic chiral metasurfaces can be realized using various top-down fabrication methods such as electron-beam lithography, ion-beam milling, or photolithography. Four typical examples of intrinsic chiral metasurfaces are shown in Figure 2. 

Figure 2a(i) shows intrinsic chiral metasurfaces with unit cells of 810 nm periodicity, composed of four rectangular-shaped slits [90]. These patterns were realized by ion-beam milling a thin gold film on a sapphire substrate. Because the chiral split patterns had different responses to the two circular polarizations of the incident light, there was a clear difference between the electric field intensity maps when under RCP (Figure 2a(i)) and LCP illumination (Figure 2a(ii)) at 720 nm. This AT induces drastic CD peaks, which are red-shifted proportionally to length of the slits, as represented in Figure 2a(iv). 

In 2017, Sun’s group suggested that chiral arrays composed of achiral nanoholes could be employed for intrinsic chiral systems (Figure 2b) [91]. Two metasurfaces were fabricated with ultra-thin gold films, which were perforated using the focused ion beam technique. For each metasurface, seven nanoholes were separated with a gap size g. In the first metasurface, the seven holes had a g of +20 nm (Figure 2b(i)). In contrast, in the second metasurface, the holes had a g of −20 nm, so the holes overlap each other (Figure 2b(ii)). This overlapped geometry brought out multiple apexes on the metasurface which became chiral hot spots that generated the remarkable chiral response, as shown in Figure 2b(iii). The period of the unit cell affected the intensity, the location of the OA peak, and also the CD peak (Figure 2b(iv)).

In 2018, Capasso’s group demonstrated dielectric gammadion nanostructures as a way to obtain near-unity transmission and CD with normally incident RCP and LCP light (Figure 2c(i)) [92]. The TiO_2_ gammadion patterns with a periodicity of 500 nm were on top of a TiO_2_ thin film and glass substrate (Figure 2c(ii)). This metasurface was optimized by considering higher-order multipoles from electric and magnetic dipoles to the magnetic octupole. The experimental result of the zeroth-order transmittance showed a significant difference between RCP and LCP incident light in the visible range (Figure 2c(iii)). At the target wavelength of 540 nm, a CD of approximately 80% was reported (Figure 2c(iv)).

Figure 2d shows an example of chiral structures that are fabricated using focused-ion beam lithography [93]. The monocrystalline silicon layer was epitaxially grown on a sapphire substrate, and an oxide glass-like coating was obtained through high-temperature annealing (Figure 2d(i)). As shown in Figure 2d(ii), the metasurface had 4-fold rotational symmetry, and the curved grooves brought out circular-polarization-dependent absorption. The optical properties including absorption, transmission, CD, and optical activity were linked, and Figure 2d(iii) demonstrates the theoretical and experimentally measured OA spectra. When the wavelength was near 755 nm, the values in the OA plot changed drastically, due to an antiresonance point. At that point, the transmittance difference between LCP and RCP light was significantly large and the CD also showed a sharp resonant peak. 

### 3.2. Extrinsic Chiral Metasurfaces

Extrinsic chirality, so-called pseudo-chirality, uses the chiral response from achiral and homogeneous nanostructures to produce chiral responses [72,94,95]. The chiral response is negligibly small at normal incident illumination. However, when the angle becomes oblique enough, the chirality-dependent signal gets stronger, allowing it to be easily detected. Four significant examples of extrinsic chiral metasurfaces are presented. 

**Figure 3:**
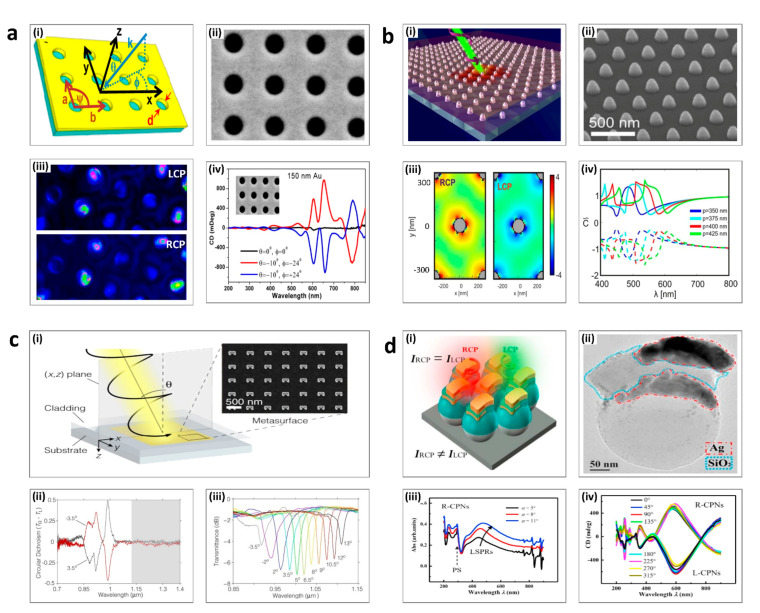
Extrinsic chiral metasurfaces: (**a**) Circular nanoholes; (i) Schematic of the metasurface. (ii) SEM image. (iii) FDTD simulation results of the electric field distribution under RCP and LCP illumination. (iv) CD spectrum. (**b**) Truncated cones; (i) Schematic view of the incident circular polarized light and metasurface. (ii) SEM image. Scale bar: 500 nm. (iii) The distribution of the optical chirality factor under RCP and LCP illumination. (iv) Plot of optical chirality factor depending on wavelength. (**c**) Split rings; (i) Schematic view of the input light and nanostructures, and SEM image. Scale bar: 500 nm. (ii) CD spectrum (iii) The transmittance with respect to incident angle. (**d**) Complex multilayer metasurface. (i) Schematic of the nanostructures. (ii) SEM image. Scale bar: 50 nm (iii) Absorption spectrum. (iv) CD spectrum depending on the tilting angle (**a**) is adapted from reference [99]. (**b**) is adapted from reference [100]. (**c**) is adapted from reference [72]. (**d**) is adapted from reference [101].

In 2012, Markovich’s group investigated the nanohole arrays in an Au film which demonstrated strong chiroptical properties by using the excitation of surface plasmon polaritons (Figure 3a(i)) [96]. The direction of the oblique incident light is defined with “ψ “ and “*θ*” with respect to normal incidence. The unit cell is a square with spacing a = b = 530 nm and the diameter of the nanohole d = 250 nm (Figure 3a(ii)). Figure 3a(iii) represents the electric field distribution as calculated using FDTD simulations, which shows that the excitation is different with respect to the different circular polarization incidence. This extrinsic chiral metasurface showed a stronger CD signal under oblique incidence (the red and the blue lines) compared to normal incidence as presented in Figure 3a(iv). 

Periodic arrays of plasmonic nanomaterials have also been demonstrated as extrinsic chiral metasurfaces. For example, in 2019, Pirruccio’s group demonstrated achiral periodic aluminum (Al) nanoparticles on a thin layer of polymethyl methacrylate (PMMA) (Figure 3b(i)) [97]. As shown in Figure 3b(ii), the truncated cones with a height of 150 nm were arranged in a triangular unit cell. The optical chirality factor, C, is defined using the following equation.

(6) C=− 12ϵ0ωIm[E→ *r→·B→r→]

$\epsilon_{0}$ is the dielectric permittivity of free space, and $\vec{E}\left(\vec{r}\right)\;\mathrm{and}\vec{B}\left(\vec{r}\right)$ are the complex electric and magnetic field vectors at position *r*, respectively. Figure 3b(iii) represents C in the XY plane at z = 150 nm and confirmed that the sign of C is opposite under LCP and RCP incidence, and that the highest magnitude is induced under LCP incidence. Because the metasurfaces exploit the excitation of collective optical resonances to produce the chiroptical responses, the peaks of super chirality (C>1) are places around surface lattice resonances points (Figure 3b(iv)). 

Metasurfaces consisting of Au split ring resonators (SRRs) are another example of periodic plasmonic extrinsic chiral metasurfaces (Figure 3c(i)) [72]. The off-axis incident illumination can be decomposed into s- and p-polarization components with respect to angle *θ*. This induces diffractive coupling of localized plasmons, which causes handedness-dependency of excitations of the lattice surface modes around the SRRs. Figure 3c(iii) shows the distinct difference of the CD spectrum when *θ* is +3.5° (black line) and −3.5° (red line). The differences were remarkable in the unshaded part, especially in the near-infrared regime from 0.85 μm to 1 μm. As shown in Figure 3c(iv), the wavelength of the maximum transmittance can be modulated by around 200 nm by changing the angle of incidence of the light, so this metasurface can be utilized as a tunable polarization spectral filter.

Metamaterials composed of more complicated three-dimensional structures can be fabricated due to the progress of advanced nanofabrication technologies. Figure 3d shows an example of the glancing angle deposition fabrication method [98]. Each chiral plasmonic nanostructure (CPN), which consists of polystyrene beads, silver (Ag), and silicon dioxide (SiO_2_), is arranged in a hexagonally close-packed structure (Figure 3d(i–ii)). The areas and width of the Ag and SiO_2_ layers are modulated by the deposition tilting angle (*α*), which is related to the geometric shadow effect. Therefore, the localized surface plasmon resonance mode is shifted with respect to an increment of ‘*α*’, which is shown in Figure 3d(iii). The azimuthal angle of deposition for the final Ag layer determines the structures response to RCP and LCP incident light, with a significant CD signal at the wavelength = 600 nm (Figure 3d(iv)). 

## 4. Applications of Chiral Metasurfaces

Conventional passive metasurfaces have a fundamental limitation in that, once a device is fabricated, its optical responses are defined and cannot be reconfigured. To overcome this, multiplexing metasurfaces have been actively researched. The multiplexing metasurfaces using polarization states of the light have not been achieved easily due to the lack of polarization-sensitive materials. Birefringent metasurface with superpixel meta-atom has been proposed to demonstrate polarization multiplexing [99,100,101]. However, it has a limitation of high-diffraction and crosstalk in the far-field.

Chiroptical properties can be used to achieve multiplexing metasurfaces due to their independent responses to the two circular polarization states. Many applications of chiroptical metasurfaces have been demonstrated. In the following section, we discussed three prospective applications of chiroptical metasurfaces: metaholograms, metalenses, and sensing and detection.

### 4.1. Metaholograms

Metaholograms reconstruct the encoded amplitude and the phase information in the metasurface to produce holographic images. Chiral metaholograms can encode more than one hologram in a single metasurface, and thus have drawn attention as a way to overcome the conventional limitation of passive metaholograms. Chiroptical metaholograms can achieve polarization-sensitive multiplexing by using polarization as an additional degree of freedom (Table 1).

One technique to incorporate multiple holograms in a single metasurface utilizing AT is by compensating for the retardation phase *α*(*x*,*y*). The second phase *β*(*x*,*y*) should be achieved by Pancharatnam–Berry PB phase, which relies on the rotation of unit elements. If the phase distribution of two independent holograms for different wave-polarizations (*ϕ_LCP_*, and *ϕ_RCP_*) is known, this retardation can be calculated using the procedure defined below.

(7) $$\phi_{LCP}=\alpha \left(x,y\right)+2\beta \left(x,y\right)$$

(8) $$\phi_{RCP}=\alpha \left(x,y\right)-2\beta \left(x,y\right)$$

From Equation (8) we can find the value of geometric phase *β* in terms of the phase distribution and retardation phase as given in Equation (9).

(9) $$\beta \left(x,y\right)=\frac{\alpha \left(x,y\right)-\phi_{RCP}}{2}$$

Upon substituting Equation (9) into Equation (7), the value of retardation can then be found as:

(10) $$\alpha \left(x,y\right)=\frac{\phi_{lcp}+\phi_{RCP}}{2}$$

where *β* is given by Equation (11).

(11) $$\beta \left(x,y\right)=mod\left[\frac{\phi_{LCP}-\phi_{RCP}}{4},2\pi \right]$$

The retardation phase can be achieved by selecting multiple unit elements for the design. Each selected element should provide the additional retardation phase depending on its geometry. The geometric phase can be attained with the help of rotation of the structures. This type of scheme generates low losses and high efficiency because the entire metasurface is utilized for both images.

**Figure 4:**
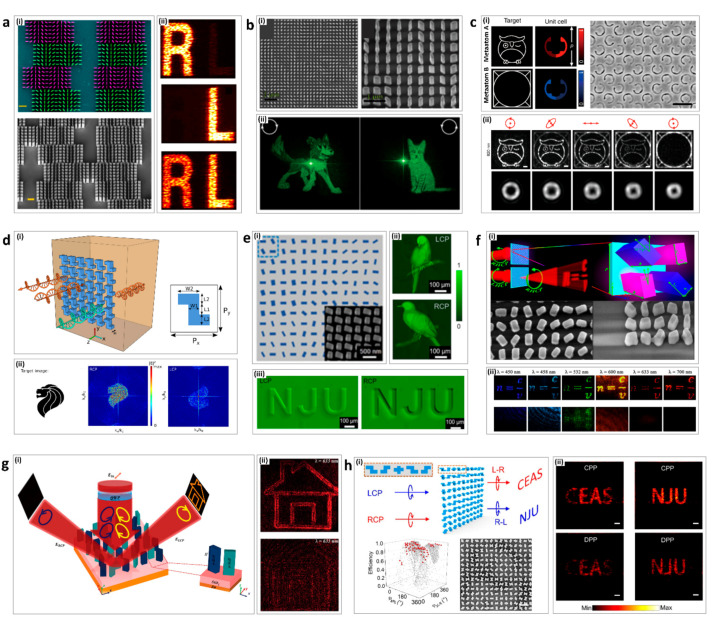
Chiroptical metaholograms: (**a**) Chiral binary metaholograms using detour phase; (i) A false-colored SEM image of the four pixels of the hologram (top) and tilted view SEM image (bottom). (ii) Images in the +1 diffraction order generated by the chiral hologram under different incident polarization, RCP, LCP, and LP, respectively, from top to bottom. (**b**) Independent metaholograms from the circular polarization states; (i) SEM images of the chiral metahologram from the top view (left) and tilted view (right). (ii) An image of a dog or cat is projected for RCP (left) or LCP (right) incidence, respectively. (**c**) Chiral metahologram with intrinsically chiral plasmonic stepped nanoapertures; (i) Illustration of the chiral metahologram merging two types of meta-atom A and B (left). SEM image of the fabricated chiral hologram (right) (ii) Reconstructed images for the chiral metahologram (top) and the spin-controlled hybrid superposition of the OAM modes (bottom) for RCP, right elliptical polarization (REP), linear polarization (LP), left elliptical polarization (LEP), and LCP. (**d**) All-dielectric planar chiral metasurface with significant AT; (i) Illustration of the chiral metasurface with significant transmission difference to LCP and RCP with Z-shape resonators. (ii) The target image for the 2D hologram (left), the generated hologram under RCP (middle) and LCP (right). (**e**) Independent amplitude control of arbitrary orthogonal states of polarization; (i) Schematic diagram of metasurface array. (ii,iii) Experimentally captured optical images of the metasurfaces. (**f**) Planar achiral metasurface for optical spin isolation; (i) Schematic of the designed supercell in the form of an array (top) and SEM images of metasurface (bottom). (ii) Captured images of the holograms at different wavelengths. (**g**) Simultaneous optical spin conservation and spin isolation in chiral metasurface; (i) The concept of the chiroptical effect of simultaneous spin conservation and spin isolation. (ii) Captured images from the holograms under LCP (top) and RCP (bottom) illumination. (**h**) Metasurfaces with planar chiral meta-atoms; (i) Illustration of chiral meta-atoms for wave manipulation (top), simulated diffraction efficiency distribution with the meta-atoms (bottom-left), and SEM image of the metasurface (bottom-right). (ii) Experimental results with RCP (left) and LCP (right) illumination on the CPP (chiral meta-atoms and PB phase) method (top) and DPP (dynamic phase and PB phase) method (bottom). (**a**) is adapted from reference [102]. (**b**) is adapted from reference [103]. (**c**) is adapted from reference [104]. (**d**) is adapted from reference [105]. (**e**) is adapted from reference [106]. (**f**) is adapted from reference [107]. (**g**) is adapted from reference [108]. (**h**) is adapted from reference [109].

In 2016, Capasso’s group achieved high efficiency (75%) broadband chiral holograms [102]. By integrating detour phase with geometric phase, the chiroptical metahologram projected two different images depending on the handedness of the incident circularly polarized light (Figure 4a(i)). An image of the letter ‘R’ is shown for RCP incidence and ‘L’ for LCP, while both ‘R’ and ‘L’ appear under linear polarized illumination (Figure 4a(ii)).

**Table 1:** Comparison of chiral metahologram.

Reference	Materials	Principle	Wavelength (nm)	Function
[105]	Amorphous silicon	Detour phase + Geometric phase	460–1800	Independent hologram (phase modulation)
[106]	Titanium dioxide	Propagation phase + Geometric phase	532	Independent hologram (phase modulation)
[107]	Gold	Stepped nanoaperture	700–1000	Independent hologram (phase modulation)
[108]	Germanium	Z-shape resonator	1655, 2000	Hologram with large CD and AT over 0.8
[109]	Titanium dioxide	Propagation phase + Geometric phase	550	Independent hologram (amplitude modulation)
[110]	Hydrogenated amorphous silicon	Superpixel of two nanofin types	450–700	Spin isolation
[111]	Hydrogenated amorphous silicon	Superpixel of two nanofin types	633	Spin isolation
[112]	Amorphous silicon	C2-symmetric meta-atom	980–1400	Independent phase modulation

In 2017, Capasso’s group demonstrated chiral holograms that enable independent phase control of arbitrary orthogonal polarization states [103]. By combining the propagation and geometric phase, two arbitrary and independent phase profiles can be imposed on a single layer of metasurface (Figure 4b(i)). Unlike previous polarization multiplexing holograms, this chiral hologram can work for any two orthogonal polarization states. Images of a cat for LCP and a dog for RCP are encoded independently in the chiral metasurface without any crosstalk (Figure 4b(ii)). Recently, Rho’s group demonstrated stimuli-responsive dynamic metaholograms by integrating liquid crystals with the chiral metaholograms [110]. 

In 2018, Gao’s group demonstrated a chiral geometric metasurface with intrinsically chiral plasmonic stepped nanoapertures [104]. The chiral metasurface consisted of superpixels with two independently designed meta-atoms of the two enantiomers (meta-atom A and meta-atom B), and thus showed both a large CD and a large cross-polarization ratio (CPR) in transmission (Figure 4c(i)). Under one state of circularly polarized light, meta-atom A allowed, while meta-atom B disallowed, the transmission. Using these properties, two images can be encoded into two independent meta-atoms, and a crosstalk-free multiplexing metahologram can be demonstrated. Under RCP (LCP) illumination, only meta-atom A (B) was functional, and images of an “owl (window)” were generated (Figure 4c(ii)). The chiral metasurface also modulated the orbital angular momentum mode from l = 3 for RCP to l = 1 for LCP (Figure 4c(iii)).

In 2018, Hong’s group theoretically demonstrated a planar chiral metahologram with Z-shape resonators that break the in-plane mirror symmetry [105]. The chiral metahologram showed high CD and AT of over 0.8, high conversion efficiency, and low loss by utilizing high refractive index germanium Z-shape resonators (Figure 4d(i)). For the simulations of chiral metaholograms, the head of a lion was used as the target image that is produced with high conversion efficiency for RCP illumination, while the amplitude of the hologram was greatly reduced, meaning that the target image disappeard under LCP illumination (Figure 4d(ii)).

In 2020, Xu’s group demonstrated new chiral metasurfaces and verified the relation between the amplitude profiles of light and orthogonal polarization states [106]. Unlike previous research which only modulated the phase profiles, two arbitrary and independent amplitude profiles can be encoded in chiral metasurfaces by combining the geometric phase and propagation phase (Figure 4e(i)). Under LCP illumination, the letters “NJU” present a stereoscopic convex effect, while the pattern of the letters “NJU” show a different concave effect under RCP light (Figure 4e(ii)). In addition, the chiral metasurface also can demonstrate two independent images for each of orthogonal circular polarization state (Figure 4e(iii)). 

In 2020, Rho’s group demonstrated a metahologram with chiroptical effects of simultaneous CCD and AT with achiral nanofins [107]. The chiral metahologram consisted of two types of nanofins in each superpixel. LCP transmission from both nanofins destructively interfered while RCP transmission from both nanofins constructively interfered. Therefore, the chiral metahologram achieved a circular CD of 55% and AT of 58% at 633 nm, resulting in the perfect absorption of LCP light, while 71% of RCP light was converted to LCP to make a transmissive type hologram (Figure 4f(i)). Moreover, the metahologram had a broadband working range in the visible regime (500–800 nm), with high AT and CD (Figure 4f(ii)). 

In 2021, Rho’s group demonstrated a reflective type of chiral metahologram that achieved both spin conversion and spin isolation via two types of meta-atoms in a superpixel [108]. The two meta-atoms make superpixels and had the same relative angle of two meta-atoms, but different angles of the superpixels. If the relative angle is π/4, the chiral metahologram theoretically reflects 72% of LCP light and produces a hologram, while suppressing 98% of RCP light (Figure 4g(i)). Under LCP light, the hologram of a “house” appeared with constructive interference of LCP light, whereas under RCP light, no visible image was produced with the destructive interference of RCP light (Figure 4g(ii)). 

In 2021, Li’s group demonstrated a new type of planar chiral meta-atoms for circularly polarized light [109]. The C2-symmetric meta-atoms had mirror symmetry and n-fold (n > 2) rotational symmetry. Unlike conventional chiral meta-atoms that use PB phase, the C2-symmetric meta-atoms themselves lead to a phase shift for different circular polarized states of light, therefore there is no need to rotate the meta-atoms to modulate the phase (Figure 4h(i)). In addition, the chiral metahologram consisting of C2-symmetric meta-atoms has a broadband operating wavelength range from 980 nm to 1200 nm, while achieving a high efficiency of over 74%. The chiral metahologram can produce two different holograms as the spin of the circularly polarized light is changed. Two images of “CEAS” and “NJU” were chosen to verify the chiral metahologram. These chiral meta-atoms demonstrated the holographic images with higher performances than those created with conventional multiplexing meta-atoms (using dynamic phase and PB phase) (Figure 4h(ii)). 

### 4.2. Metalenses

Metalenses have received great attention in order to overcome the limitations of conventional diffractive lenses, which are usually bulky and heavy. However, the limitations of passive metalenses, such as fixed focal lengths, chromatic aberration, etc., hinder their practical application. To overcome these limitations, multiplexing metalenses have been actively researched. In the following, we introduced chiral multiplexing metalenses (Table 2).

**Figure 5:**
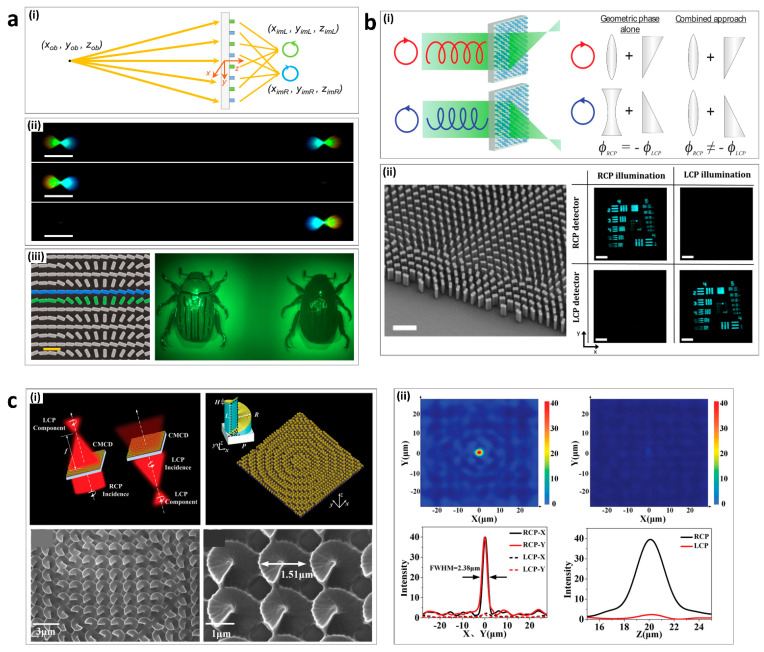
Chiroptical metalenses: (**a**) Multispectral chiral imaging with a metalens; (i) Schematic diagram illustrating the imaging principle of the chiral metalens. (ii) Two images (top), left image (middle), and right image (bottom) formed by the chiral metalens into the field-of-view of a color camera. (iii) SEM image of the metalens (left) and the chiral metalens forms two images of the beetle on the color camera. (**b**) High-efficiency chiral metalens; (i) Chiral metalens principle. (ii) SEM image of metalens. (iii) RCP and LCP images taken of the 1951 USAF resolution test chart with the chiral metalens under RCP and LCP illumination. (**c**) Chiral metalens with helical surface arrays; (i) Schematic of a function of a chiral metalens (top) and SEM images of chiral metalens (bottom). (ii) Focusing performance of the designed transmitting chiral metalens. Intensity distribution (top) and line scan of the intensity distribution (bottom) for RCP (left) and LCP (right). (**a**) is adapted from reference [111]. (**b**) is adapted from reference [112]. (**c**) is adapted from reference [113].

In 2016, Capasso’s group demonstrated a metalens with chiral imaging, which simultaneously formed two images with the opposite-handedness of circularly polarized light within the same field of view [111]. The chiral metalens consisted of two types of meta-atom (green and blue) (Figure 5a(i)). Green meta-atom was designed to focus RCP light while the blue meta-atom was designed to focus LCP light. For an object located at (*x_ob_*, *y_ob_*, *z_ob_*), the RCP image of the object was focused at (*x_imR_*, *y_imR_*, *z_imR_*) and the LCP image of the object was focused at (*x_imL_*, *y_imL_*, *z_imL_*). The chiral metalens can simultaneously focus and disperse two identical broadband beams with linear polarization (Figure 5a(ii)). For LCP (RCP), the right (left) beam disappeared while the intensity of the left (right) beam increased (Figure 5a(ii)). The CD of the chiral metalens can be mapped with the chiral beetle, *Chrysina gloriosa*, which has high reflectivity of LCP (Figure 5a(iii)). 

**Table 2:** Comparison of chiral metalens.

Reference	Materials	Principle	Wavelength (nm)	Function
[114]	Titanium dioxide	Superpixel of two nanofin types	532, 488, 620	Multispectral chiral lens
[115]	Titanium dioxide	Superposition of lens and wedge phase profiles	470–650	Overcome 50% efficiency trade-off and achieve 70% efficiency
[116]	Gold	Gradient helical meta-atom	3–5 μm	Chiral metalens of circular dichroism

The phase difference (*φ_d_*) from object to image for such a system can be calculated for both RCP and LCP images (Equations (12) and (13)).

(12) $$\varphi_{d-r}\left(x_{R},y_{R},z_{R}\right)=\frac{2\pi }{\lambda_{d}}\left(\Delta D_{ob-r}+\Delta D_{im-r}+f_{r}\right)$$

(13) $$\varphi_{d-l}\left(x_{L},y_{L},z_{L}\right)=\frac{2\pi }{\lambda_{d}}\left(\Delta D_{ob-l}+\Delta D_{im-l}+f_{l}\right)$$

where *r* represents RCP, and *l* represents LCP. The terms *ob* and *im* represent object and image, respectively. The ΔD term represents the distance from a reference point on metasurface ((*x_R_*, *y_R_*, *z_R_*) for RCP and ((*x_L_*, *y_L_*, *z_L_*) for LCP) to either the object or image for different polarizations. These terms are further defined below.

(14) $$\Delta D_{ob-r}=\sqrt{{\left(x_{R}-x_{ob}\right)}^{2}+{\left(y_{R}-y_{ob}\right)}^{2}+{\left(z_{R}-z_{ob}\right)}^{2}}$$

(15) $$\Delta D_{im-r}=\sqrt{{\left(x_{R}-x_{im-R}\right)}^{2}+{\left(y_{R}-y_{im-R}\right)}^{2}+{\left(z_{R}-z_{im-R}\right)}^{2}}$$

(16) $$\Delta D_{ob-l}=\sqrt{{\left(x_{L}-x_{ob}\right)}^{2}+{\left(y_{L}-y_{ob}\right)}^{2}+{\left(z_{L}-z_{ob}\right)}^{2}}$$

(17) $$\Delta D_{im-l}=\sqrt{{\left(x_{L}-x_{im-L}\right)}^{2}+{\left(y_{L}-y_{im-L}\right)}^{2}+{\left(z_{L}-z_{im-L}\right)}^{2}}$$

where *f* is the sum of the focal lengths of the object and image, the term f can be written as follows:

(18) $$f_{r}=f_{ob}+f_{im-R}$$

(19) $$f_{l}=f_{ob}+f_{im-L}$$

where,

(20) $$f_{ob}=\sqrt{x_{ob}+y_{ob}+z_{ob}}$$

(21) $$f_{im-R}=\sqrt{x_{im-R}+y_{im-R}+z_{im-R}}$$

(22) $$f_{im-L}=\sqrt{x_{im-L}+y_{im-L}+z_{im-L}}$$

Substituting the values of distances and focal lengths in Equation (12) and Equation (13), the phase compensated by each of the meta-atoms can be attained. Since PB phase is being employed, the rotation angle (*θ_R_* and *θ_L_*) of each meta-atom (for RCP and LCP) should be half of the required phase.

(23) $$\theta_{R}=-\frac{\varphi_{d-r}\left(x_{R},y_{R},z_{R}\right)}{2}$$

(24) $$\theta_{L}=-\frac{\varphi_{d-l}\left(x_{L},y_{L},z_{L}\right)}{2}$$

In 2018, Capasso’s group demonstrated a high-efficiency chiral metalens, which enabled independent focusing for each of the circularly polarized states of light without a 50% efficiency trade-off [112]. The conventional metalens focuses LCP (RCP) light while incident RCP (LCP) light diverges. This results in more than a 50% efficiency loss. In addition, diverging light causes noise and lowers the quality of imaging. This chiral metalens overcame these limitations to achieve a 70% efficiency by combining the phase profiles of a lens and wedge (Figure 5b(i)). The target image was produced by the chiral metalens under different circular polarizations (Figure 5b(ii)). 

In 2019, Wang’s group demonstrated a chiral metalens with helical meta-atoms that operated in the mid-infrared region [113]. The distributed gradient helical surface meta-atoms were covered in a gold film, resulting in chiral imaging with high circular polarization dichroism (Figure 5c(i)). By rotating each helical meta-atom along the radial direction, geometric phase can be used to modulate the phase response. In the chiral metalens of circular polarization dichroism, one-handedness of circularly polarized light was focused in transmission, while the other was reflected (Figure 5c(ii)). The full width at half maximum at the focal spot of the metalens of 2.38 μm is close to the diffraction limited performance of 1.76 μm. 

### 4.3. Bio-Sensing and Detection

Chiral sensing and detection rely on the fact that chiral molecules enhance the near field when they are in the vicinity of a chiral metasurface (Table 3). Therefore, there is a huge potential for its use in the biomedical and pharmaceutical industries, where different enantiomers have critical differences in some of their chemical and physical properties, and sometimes even in their toxicity levels [114]. The designed metasurfaces can distinguish between different enantiomers of proteins and antibodies. It was also observed that an increase in the size of the unit-cells of a metasurface could lead to better sensing, especially if nanorods are used, due to their larger surface area. Chiral metasurfaces can also help in sensing DNA and cancer biomarkers, which make chiral metasurfaces a very exciting tool in the field of CD spectroscopy [115].

**Figure 6:**
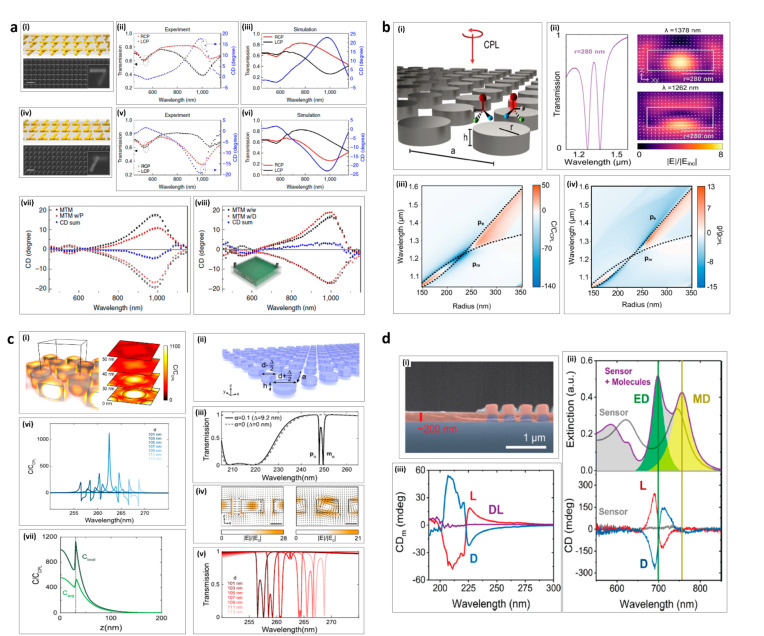
Chiroptical bio-sensing and detection: (**a**) Chirality-based detection of enantiomers using plasmonic twisted gold metamaterials; (i) Metamaterial enantiomer with +60 twist. (ii) Experimental results of enantiomer with +60 twist. (iii) Simulated results of enantiomer with +60 twist. (iv) Metamaterial enantiomer with -60 twist. (v) Experimental results of enantiomer with -60 twist. (vi) Simulated results of enantiomer with -60 twist. (vii) CD results from metamaterial with and without protein. (viii) CD results from metamaterial with and without drug. (**b**) High refractive index chiral sensing using disks; (i) Schematic of the design. (ii) Transmission image and field enhancement from the design. (iii) Enhancement in optical chirality. (iv) Enhancement in Kuhn’s dissymmetry. (**c**) Ultraviolet spectroscopy employing dielectric metasurfaces; (i) Field enhancement in the design at various z-planes. (ii) Schematic of the design and visual representation of spacing between the disks. (iii) Transmission with and without difference in diameters between two disks. (iv) Field enhancement in the design. (v) Transmission with various diameters of the disk. (vi) Maximum optical chirality achieved for the design. (vii) Height-dependent local and average chirality attained for the design. (**d**) Chiral sensing using dielectric resonator; (i) Scanning electron micrograph image of the fabricated device. (ii) Experimentally measured values of CD represented by Cd_m_ for L-, D-, and a racemic mixture of the phenylalanine coating. (iii) Experimental results of extinction (top) and CD (bottom) results of the sensors with and without coating. (**a**) is adapted from [116]. (**b**) is adapted from [86]. (**c**) is adapted from [117]. (**d**) is adapted from [118].

In 2016, Alu’s group proposed a plasmonic metamaterial design that consisted of two metasurfaces of twisted gold rods stacked with a separation of 80 nm [116]. A pair of such metamaterials in the form of enantiomers with a twist of +60 and −60 were fabricated as shown in Figure 6a(i,iv). Both enantiomers achieved almost the same optical properties, but with polarization dependency, producing a large CD. The enantiomer with +60 twist gave a positive value of CD when CD was taken to be RCP-LCP (Figure 6a(ii–iii)). On the other hand, the enantiomer with −60 twist had a negative value of CD (Figure 6a(v–vi)). These results were verified by simulations and experiments. For sensing purposes, two analytes of protein (concanavalin A at a concentration of 1 mg ml^−1^) and an anticancer drug (Irinotecan hydrochloride at a concentration of 1 mg ml^−1^) were spin-coated on the metamaterial as shown in Figure 6a(vii–viii), respectively. The CD summation (shown in the blue curve) represents the handedness of the analytes. If the CD summation showed a negative bend, this meant that the analyte was right-handed, and vice versa. This application drastically increased the sensitivity to molecular chirality, allowing the detection of approximately 55 zeptomoles of molecules in the imaging area.

In 2019, Dionne’s group proposed a metasurface consisting of high refractive index dielectric disks that demonstrated enhancements in the optical chirality and Kuhn’s dissymmetry factor [85] (Figure 6b(i)). The transmission and field enhancements are shown in Figure 6b(ii). We can observe a 138-fold local enhancement in the optical chirality and a 15-fold local enhancement in Kuhn’s dissymmetry factor by modulating the radius of the disks, as presented in Figure 6b(iii–iv). These enhancements in optical chirality and Kuhn’ dissymmetry are found to be entirely left- or right-handed over the whole metasurface.

In 2019, Dionne’s group also proposed a high-quality factor diamond metasurface, operating in the ultraviolet (UV) regime that enhances the optical chirality by up to 3 orders of magnitude [117] (Figure 6c(i)). The proposed design utilized a biperiodic dielectric disk metasurface, where the difference in diameters between the two disks induces a chiral response (Figure 6c(ii)). The transmission results for a 9.2 nm difference in disk diameters is shown in Figure 6c(iii), where two resonances from the electric (p*_α_*) and magnetic modes (m*_α_*) are observed. These resonances are also observed to be dipole-like (Figure 6c(iv)). The transmission from various disk diameters is presented in Figure 6c(v), and it was observed that, when the difference in diameters was approximately 10%, the optical chirality enhancement exceeded 1000-fold at the radial face of the metasurface. However, the average optical chirality enhancement up to 40 nm away from the metasurface increased beyond approximately 100-fold (Figure 6c(vii–viii)). 

In 2020, Quidant’s group proposed a metasurface sensor based on amorphous silicon cylinder nanorods (Figure 6c(i)) that were used to measure the chirality induced by the two enantiomers (L-, and D-) of phenylalanine amino acid and their racemic mixture [118]. This metasurface was designed to achieve high enhancements in the chiroptical response without having any CD response of its own (Figure 6c(ii–iii)). The design distinguished between the two enantiomers effectively, with an around 5-fold enhancement in the molecular CD, mostly due to the electric dipole response. This amount of enhancement was more significant than previously reported in plasmonic sensors [119]. 

For the purpose of detecting and sensing proteins, an important work included passive and active devices with metal-dielectric-metal layers of Au-SiO2-Au (where the active device was loaded with graphene) for sensing within 5000–8400 nm [120]. Similarly, for the detection of fructose, chlorpyrifos methyl, and Gamma-aminobutyric acid (GABA), another MDM based design had a gold patch array with an Au-Polyimide-Al and graphene coating on top, and a design of gold nano dipoles, which were proposed to detect these compounds in THz regime [121,122].

Since chiral sensing and detection plays a major role in detection of diseases, a design of nanopost array employing Si, was used to detect cancer within 1520–1580 nm [123]. Similarly, a design of concentric ring resonators of gold with polyimide substrate was employed to detect oral cancer from 0.5–1.5 THz regime [124]. Another prominent work detected proteins and HIV virus using plastic-templated (polycarbonate) tunable metasurface with periodic metal-dielectric layers of gold, silver, and titanium from 500–800 nm [125].

**Table 3:** Comparison of chiral sensing and detection.

Reference	Materials	Design	Wavelength (nm)/Frequency (THz)	Molecule/Compound Sensed
[116]	Gold	Twisted gold stacks	750–1150 (nm)	Two analyses of protein and anticancer drug
[77]	Silicon	Disks	150–350 (nm)	Thiocamphor
[118]	Silicon	Nanocylinders (disks)	600–1000 (nm)	Racemic mixture of phenylalanine amino acid
[125]	Gold, Silver, Titanium, Polycarbonate	Plastic-templated tunable metasurface with periodic meta-dielectric layers	500–800 (nm)	Proteins and viruses (prominently HIV)
[120]	Gold, SiO_2_, Gold	Passive device with MDM layers of Au nanoantenna, SiO_2_, Au back mirror	~5000–8400 (nm)	Protein
[120]	Gold loaded with graphene, SiO_2_, Gold	Active device with MDM layers of Au nanoantenna loaded with graphene, SiO_2_, Au back mirror	~5000–8400 (nm)	Protein
[123]	Silicon	Nanopost array (disks)	1520–1580 (nm)	Cancer
[121]	Aluminum, Polyimide, Gold, Graphene	Gold patch array with MDM layers (Au-Polyimide-Al) and graphene coating on top	0.8–1.1 (THz)	Fructose and chlorpyrifos methyl
[124]	Gold, Polyimide	Concentric ring resonators	0.5–1.5 (THz)	Oral cancer cell SCC4
[122]	Gold	Nano dipoles	30–120 (THz)	Gamma-aminobutyric acid (GABA)

## 5. Conclusions

Chiral metasurfaces have been used to enhance chiroptical signals with the interaction between the local fields of metasurfaces and chiral molecules, overcoming the limitations that exist in conventional optical spectroscopy. In this review, we have summarized the fundamental principles of chiroptical metasurfaces and have explained how they resonate with chiral molecules to enhance chiroptical signals. Furthermore, we categorized the types of chiroptical metasurface into intrinsic and extrinsic chiral metasurfaces. Finally, we introduced applications of chiroptical metasurfaces for multiplexing metaholograms, metalenses, sensors, and detectors.

Chiral metasurfaces can be applied in many fields, and have great potential for further improvement. Chiral metaholograms can overcome the limitations of passive metaholograms to demonstrate multifunctional metaholography, such as a switchable hologram, optical-spin isolation, and independent amplitude modulation for different-handedness of incident circularly polarized light. Chiral metalenses enable the simultaneous focusing of two images. Moreover, chiral metasurfaces have the potential to demonstrate multifunctional metalenses, such as multi-focal length metalenses. In addition, chiral metasurfaces can be applied to demonstrate chiral metamirrors [74], multi-mode orbital angular momentum (OAM) generators [65], OAM sensors [126], intense light sources [127,128], and color filters [129]. Apart from these applications, another significant application of chiroptical metasurfaces lies in chiral sensing [130,131], which can directly benefit biomedical and pharmaceutical industries. We believe that research in chiroptical metasurfaces will promote further improvement and integration within various different fields.

## Abbreviations

The following abbreviations are used in this manuscript:

Ag	Silver
Al	Aluminum
AT	Asymmetric transmission
CB	Circular birefringence
CD	Circular dichroism
CPN	Chiral plasmonic nanostructure
CPR	Cross-polarization ratio
EM	Electromagnetic
LCP	Left-circularly polarized
OR	Optical rotation
ORD	Optical rotatory dispersion
PMMA	Polymethyl methacrylate
RCP	Right-circularly polarize
SEM	Scanning electron micrographic
SiO_2_	Silicon dioxide
UV	Ultraviolet
